# Evaluation of Rodent Hair Tubes for Activity Indices

**DOI:** 10.3390/ani14060843

**Published:** 2024-03-08

**Authors:** Joanna Dürger, Orestis Kazasidis, Héloïse Brotier, Jens Jacob

**Affiliations:** 1 Institute for Epidemiology and Pathogen Diagnostics—Rodent Research, Julius Kühn-Institut (JKI) Federal Research Centre for Cultivated Plants, Toppheideweg 88, 48161 Münster, Germanyjens.jacob@julius-kuehn.de (J.J.); 2 Faculty of Science and Technology, University of Tours, Parc Grandmont, 31 Av. Monge Bâtiment G, 37200 Tours, France

**Keywords:** hair tube, rodent, small mammals, activity index, wildlife camera, habitat

## Abstract

**Simple Summary:**

Hair tubes provide a non-invasive method for determining the presence and activity of small mammals by evaluating hair left in hair tubes. Data can be converted into activity indices. In this study, a specially adapted program was developed to semi-automatically determine hair density as a proxy of activity. Adhesive tape with hair from a field experiment was processed, scanned, and analyzed by the program to obtain a quantitative measure of hair density. The validation of hair tubes with wildlife cameras in the field showed a moderate-to-strong positive correlation between hair tube data and recorded rodents. The program is simple but effective and does not require a large amount of deep learning data. Due to its reduced complexity, it facilitates error detection and fine-tuning. The use of hair tubes in combination with this program should provide an easy-to-use, non-invasive method to determine small mammal activity.

**Abstract:**

Activity indices are used to determine the presence and activity of small mammals, such as the hair index derived from the use of hair tubes. In contrast to trapping animals, hair tubes are non-invasive and less labor-intensive, and appear to be a suitable alternative in appropriate settings. We developed a method to calculate hair density semi-automatically. In addition, hair tube data were validated with field data using wildlife cameras for the small mammal community in grassland, wheat crops, and hedges to assess how well data from hair tubes match data from wildlife cameras. Adhesive tape with hair from hair tubes was processed and scanned. The resulting images were analyzed using a newly developed computer program that enables background and adhesive tape to be automatically distinguished from hair, providing a quantitative measure of hair density. Based on validation with wildlife cameras, hair tubes seem to be a suitable tool to estimate small mammal activity at the community level in several habitats. There was a moderate-to-strong positive correlation of the hair tube index with the sum of voles and *Apodemus* individuals (activity index) recorded in grasslands (Spearman’s correlation coefficient 0.43), hedges (0.79), and wheat (0.44). The newly developed computer program allows the automatic calculation of hair density, making it easier to assess the activity of small mammals.

## 1. Introduction

For obtaining data on presence/absence, population dynamics, as well as the spatial and temporal activity of dynamics, indirect methods including hair tubes have been developed [1]. The presence and density of hair is used as a proxy for animal activity and/or abundance. Hair tubes were first used by Suckling (1978) [2] to detect small mammals in trees. Gurnell et al. (2009) [3] used hair tubes for surveying and monitoring squirrels, to examine squirrel presence as a relative index of animal numbers and to distinguish between red and grey squirrels.

Indirect methods for the detection of small mammal activity such as hair tubes are particularly useful for studying the presence/absence or abundance of species or small mammal communities and their distribution [4,5,6]. The relative abundance and even absolute abundance of populations can only be derived if the system is heavily dominated by one target species. If this is not the case, genetic analyses are needed to identify species or individuals, or hair needs to be examined morphologically for species identification. Furthermore, they are helpful in surveying rare or elusive species because they do not interfere with their activity [7] and can be applied at a large spatial scale. Unlike for live traps, the frequent checking of hair tubes is not required in these analyses [8], making them less labor-intensive and less expensive [1,9,10].

Sampling hair with hair tubes is non-invasive because it does not involve restricting the movement, handling, or stressing the target species [11] or other animals that access the hair tube [12]. The application of hair tubes does not require particular skills in handling wild animals and does not affect their well-being [6,11]. Unlike for trapping, there is no disturbance (live trapping) or removal (snap trapping) of individuals, and therefore no potential effects on population dynamics [11]. This is similar to burrow counts, tracking plates, and eDNA, which do not require animal handling in contrast to live trapping or snap trapping, which are also used to assess the activity, presence, and/or abundance of small mammals.

Animals pass through hair tubes, which are often baited, and hair samples are collected on the attached tape to detect animal presence [2]. Hair tubes are suitable for different environments and can be adapted in size and design to suit different species [13]. They can be custom-built to detect only a specific taxon or designed to allow the collection of hair from different species [7]. For example, “panpipe” hair tubes with several tubes measuring varying diameters are used to detect small mammals of different sizes [8]. Pocock and Bell (2011) [5] developed a hair tube with a species-specific aperture size to collect hair from pygmy shrews (*Sorex minutus*). For common hamsters (*Cricetus cricetus*), tubes were placed directly in the entrances of hamster burrows [14]. There are also hair tubes that can only be triggered once to obtain a hair sample from a single individual [15]. Furthermore, the bait can be adapted to the target species [5,7,10,13]. Hair tubes can be left in the field for days or even weeks before being collected [9]. 

A limitation of hair tubes is that the number of individuals of the same species that enter the hair tubes cannot be readily assessed [9]. To obtain further information such as that on species, hair samples can be identified by hair anatomy [7,16,17] or genotyped [18]. The latter also allows a distinguishment between sexes and among individuals. The deployment of wildlife cameras can be helpful in confirming hair tube results [10,19,20,21].

Even for the simplest approach of using hair tubes as an indicator of presence/absence, there has been little previous systematic replicated work with small mammals. There is a lack of quantitative assessment of hair tube data [11]. Analyzing hair left on sticky tapes quantitatively could help to more precisely assess small mammals’ abundance based on hair tube data. This can be confirmed based on the findings of Chiron et al. (2018) [11], which show a linear relationship of rodent abundance and hair density for a duration of about 7 days.

Yellow sticky paper traps are used to sample insects, detect insect infestation, and estimate species abundance in the field or in greenhouses. Manually counting and identifying insects is a time-consuming and tedious procedure [22,23,24]. Furthermore, humans are slower in these tasks and make more mistakes than machines do [25]. Therefore, attempts are made to carry out analyses of yellow sticky paper traps automatically. For counting insects and the identification of species, sticky traps are photographed and automatically assessed [24,26,27]. Automatic image recognition programs reduce labor and costs, and support large-scale monitoring [23]. For identifying and counting pest insects in images, automatic detection programs based on deep learning are used [25]. 

So far, nothing similar exists for the analysis of hair tubes for small mammals. Digital quantitative evaluation of rodent hair tubes could be used to assess hair density to improve the use of hair tubes for activity indices of small mammals. Ideally, an automated system would also allow the determination of species from hair of the hair tubes but this approach seems unlikely to be developed soon.

We developed a computer program that allows the automatic calculation of hair density on adhesive tape with hair from hair tubes. The system was tested for reproducibility and validated on the community level with data from the field where hair tubes and wildlife cameras were used simultaneously in grasslands, wheat fields, and hedges. Using validated approaches and the automated assessment of hair density should facilitate the non-invasive determination of small mammal activity.

## 2. Materials and Methods

### 2.1. Hair Tube Design

**Figure 1:**
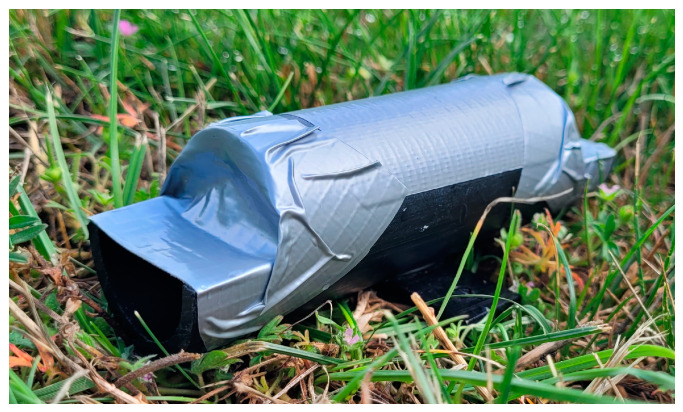
Hair tube for small mammals, based on the design reported in Chiron et al. (2018) [11]; © J. Dürger, JKI.

The hair tubes consisted of 13 cm long black polyethylene tubes with an outer diameter measuring 40 mm. The ends of the hair tubes were open to allow the passing of animals in both directions. Furthermore, the ends were cut out at a length of about 19 mm so that double-sided tape (Fermoflex, Orafol) could be applied at about half the height of the tube (about 20 mm) on either side of the tube, under which the animals entered the tube. This type of tape is durable and was sufficiently sticky for the course of the study. A larger piece of tape covered and protected the smaller piece of double-sided tape and the gap in the vertical side of the tubes (Figure 1). When small mammals passed through the tubes, they left loose dorsal guard hair on the attached tapes. The tubes were baited with about 1.0 g of peanut butter once at the beginning of the experiment. Peanut butter was chosen because it sticks well to the tube interior and is attractive to several small mammal species [10,28,29]. Adhesive tape with hair from hair tubes from the experiment, to compare the performance of hair tubes with wildlife cameras (see Section 2.4), was used for developing and testing the reproducibility of the procedure for semi-automatic hair density calculation.

### 2.2. Program for Semi-Automatic Calculation of Hair Density

**Figure 2:**
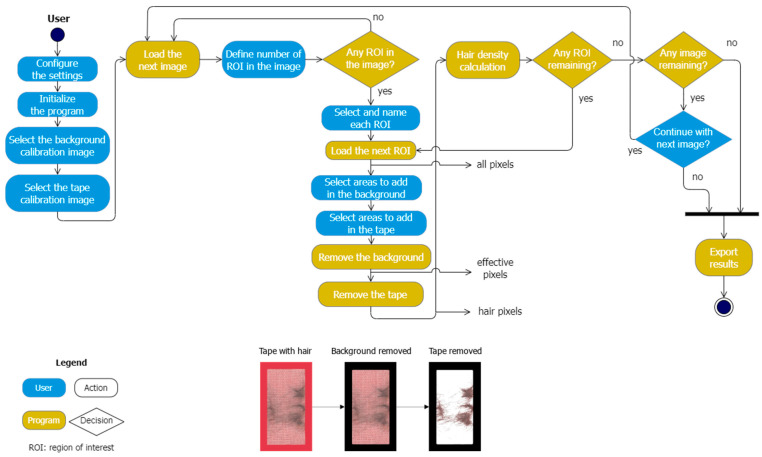
Flowchart of the program. The shape color differentiates automatic processes from expected user inputs. The rounded rectangles designate actions from either the program or the user, and the diamond shapes designate program decisions. The hair density is calculated as the ratio of hair pixels to the total tape pixels (effective pixels). ROI—region of interest; the bottom of the figure shows adhesive tape with hair, with background pixels removed by the program and tape pixels removed by the program (left to right).

The double-sided adhesive tape was manually removed from the hair tubes by carefully lifting one edge and pulling it off, taking care not to damage the tape. It was stuck to a document wallet made of transparent foil (A4) and labeled. Tapes on a red-colored background were scanned with a standard commercial scanner (Canon PIXMA MG5700, Cannon Germany, Krefeld, Germany) as TIFF files with a resolution of 600 dpi. After initialization, the program requests the user to select background pixels only (the background calibration image) and sticky tape pixels only (the tape calibration image). This is required by the program to clearly distinguish the background from the image of the sticky tape. Then, the first image of an A4 page with sticky tapes containing hair was loaded, and the number of sticky tape sections (regions) to be scanned was manually defined. Each of these regions was selected and labeled. Interfering elements such as dirt or plant particles were assigned to the background or the tape, respectively, after manual selection by the user. Finally, the program removed all background and all tape pixels in order to calculate the percentage of hair present on the tape for each region. Once all regions of interest were processed, the results with the quantitative measure of hair density (% in region of interest (ROI)) could be exported (Figure 2). 

### 2.3. Reproducibility of Results 

We developed a method for estimating the absolute error of the automatic hair density calculation, while also calculating the reproducibility of the method. We selected sticky tapes from the field data whose apparent hair density spanned a wide range, from mostly empty to high values. We used default settings in the RGB color space for the background and the tape as the calibration method. 

#### 2.3.1. Calculated Hair Density 

We repeated the automatic calculation with the program 10 times, by manually redefining the region of interest, whilst attempting to introduce variability as expected from each realistic user input (e.g., should this ragged tape edge be cut off? Can this grain of dust be ignored or should it be assigned to the tape pixels?). The arithmetic mean of the results gave the calculated value of the hair density. From the standard deviation of the results, we estimated the standard error of the mean. 

#### 2.3.2. Real Hair Density 

In the final calculation for each region, we explicitly marked all the pixels belonging in the background and in the tape, leaving only the hair pixels. This is a tedious manual process with the goal to mitigate most of the program uncertainties. The result served as a proxy for the real value of hair density. A residual overestimation is expected, because the program currently supports only rectangular region selection, which calls for the stepwise marking of tape pixels around hairs that are not parallel to the scanner axes.

### 2.4. Validation with Field Data

Hair tubes and wildlife cameras (Moultrie® M-40/M-50, Schery Revier Live, Fulda, Germany) were installed in hedges, wheat crops, and grasslands in the surroundings of Münster (51°58′ N, 7°38′ E; 39–99 m.a.s.l.) in Northwest Germany in May–July 2023. For comparability, each of these habitats bordered on rape fields. 

Five replicates per habitat were monitored in a given week, and further five habitat replicates were monitored in the following week (n = 10 replicates per habitat). In each of the 10 habitat replicates, 10 hair tubes were placed along a transect with 10 m spacing for three consecutive 24 h periods. This schedule was repeated four times within 8 weeks and resulted in n = 40 replicate measurements per habitat (10 habitat replicates × 4 repetitions = 40—these are the statistical units) based on a total of 2400 adhesive tapes examined (3 habitats × 10 replicates × 10 hair tubes × 2 sections of adhesive tape per tube × 4 repetitions = 2400). After collecting the tubes from the field, the tapes were removed and scanned to be analyzed with the program. 

The cameras were positioned to focus on one of the center tubes of each transect to monitor both ends of the hair tube. The settings were as follows: three photographs with a 10 s trigger delay, and high sensitivity. The software Agouti© (Copyright 2022, Agouti.eu, accessed on 31 July 2023) was used to count and identify small mammals of the taxon *Apodemus* (mostly *Apodemus sylvaticus*), and voles (mostly common voles (*Microtus arvalis*) and bank voles (*Myodes glareolus*)). After a picture was taken, there had to be a period of 10 min without the presence of a small mammal until the next picture was considered for analyses. This was done to minimize the probability of counting the same individual repeatedly [30].

### 2.5. Statistical Analyses

The percentage of hair detected on sticky tape from both sides of the same tube was averaged, and the mean of the values of all 10 tubes from the same transect in the same 3-day period was calculated. This resulted in one data point per sampling week per habitat replicate for the percentage of hair (40 values per habitat). These data points were paired with the data points from the associated wildlife cameras. The latter was equal to the numbers of *Apodemus* and voles that were summed to one value, resulting in a measure of rodent activity at the rodent community level. Hair tube data and data from wildlife cameras were Johnson-transformed using an algorithm to find an optimal formula with optimal parameters to normalize data distribution [31]. Spearman’s correlation test was used to test for correlations between results from hair tubes and camera traps at the rodent community level, separately for each of the three habitats.

## 3. Results

### 3.1. Program

The program was written in Python version 3.8 [32] using the libraries numpy (v. 1.24.3) [33], pandas (v. 1.3.1) [34], and matplotlib (v. 3.4.0) [35]. Image reading, processing, the manual marking of regions, and pixel counting were handled by the library opencv-python (v. 4.5.3.56) [36], using the functions imread, inRange, getStructuringElement, morphologyEx, dilate, bitwise_and, selectROI, and countNonZero, among others. The results shown in this study were obtained under Windows 10 Enterprise LTSC with an executable file created by using pyinstaller (v. 5.2) [37]. The program results were exported as an XLSX file to be further processed and visualized in Excel (Microsoft Office Professional Plus 2016).

The program works alternately in two spaces: the space of the image pixels, where manual region marking takes place and image masks are defined and applied; and the RGB color space, where thresholds are applied and color ranges are selected. For example, the background and tape pixels are firstly distinguished in the RGB color space, based on the calibration values. Subsequently working in the space of the image pixels fills remaining gaps that are caused by any deviation from the calibration values, the variance of the brightness, and the surface roughness. A red-colored background was chosen because it created the largest separation between background and tape pixels in the RGB color space.

### 3.2. Reproducibility of Results

**Figure 3:**
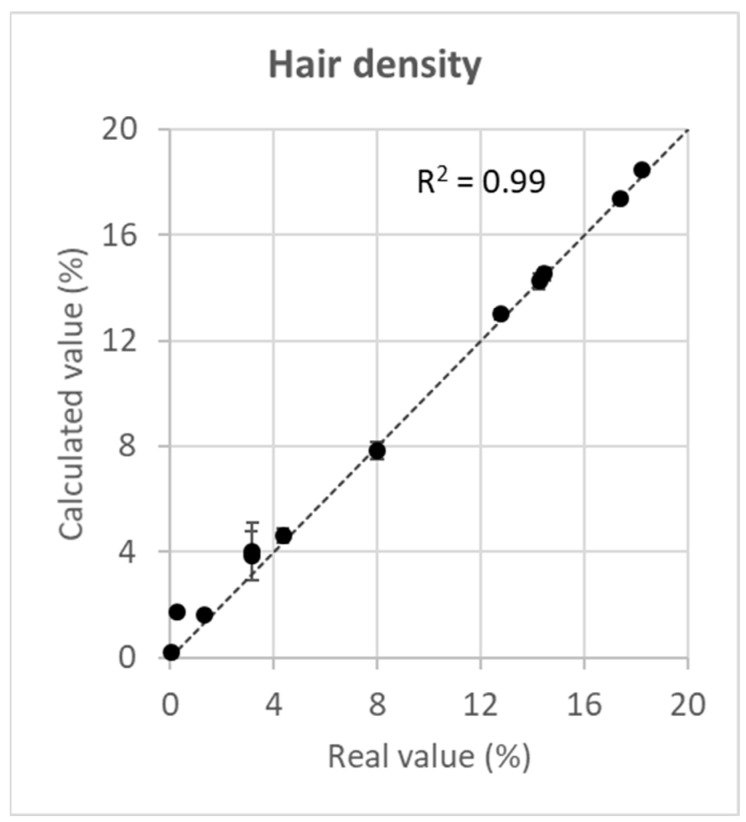
Correlation of the real value of hair density with the calculated value of hair density. The broken line reflects a perfect match of real and calculated values (real value of hair density = calculated value of hair density). Error bars are ±5 standard errors.

The deviation of the calculated values of hair density from the real values was minimal in the observed range of hair density (Figure 3, Table A1). At low hair densities of <5%, the deviation was somewhat higher (mean estimation error = 0.6) than that at higher values of >10% (mean estimation error = 0.1). The correlation of real and calculated values was positive and almost perfect (R^2^ = 0.99).

### 3.3. Validation with Field Data

Over the course of the field study, there were 1086 adhesive tapes with hair and 1314 without hair (Table A2), resulting in 43–47% of adhesive tapes with hair in the three habitats.

In the camera pictures, we identified mostly *Apodemus* (1220 sightings) and vole species (193 sightings) (Table A3). Sightings of other small mammals were of rats (22) and shrews (2) and were not considered further.

**Figure 4:**
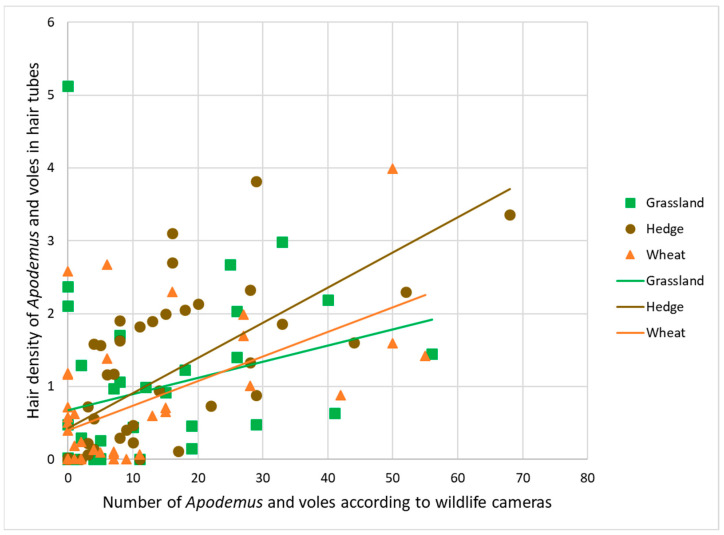
Correlation between hair tube results (hair density on sticky tape averaged per habitat replicate) and wildlife camera results (numbers of sightings of *Apodemus* and voles) in habitats grasslands, hedges, and wheat crops.

There was a statistically significant positive correlation of counts of rodents from wildlife camera images with the percentage of hair present in hair tubes in grasslands (0.43, <0.001), hedges (0.79, <0.001), and wheat (0.44, <0.001) estimated with the program (Figure 4, Table A4).

## 4. Discussion

The custom-developed computer program allows us to evaluate hair samples from hair tubes semi-automatically and to utilize the resulting quantitative measure of hair density as an activity index of small mammals. The user can analyze large quantities of adhesive tapes with hair from hair tubes quickly and accurately, resulting in work simplification for the evaluation of field trials with hair tubes. Therefore, the program allows designing an easy-to-use, non-invasive method for determining small mammal activity. The data obtained in grasslands, wheat crops, and hedges from hair tubes match the data from wildlife cameras very well on the community level.

There are numerous programs for automatic image recognition using deep learning techniques, for example to identify insects on images of sticky paper [22,38] or to recognize animal footprints [39,40]. Semi-automatic programs like ours can also be beneficial, because automating the detection of the hair density is a practical tool that makes the work easier and leads to more accurate results compared with those obtained under manual processing. This approach also results in a higher resolution of hair density data as a proxy for activity/abundance—percent values in this case—than does the use of binary information or a handful of activity/abundance classes otherwise often derived from hair tube data. There might be opportunities in the future to adapt this approach to analyze data from tracking plates, etc., in wildlife monitoring surveys.

This program is a simple but effective method compared with automatic recognition programs based on deep learning. It uses well-established traditional techniques of digital image processing and does not require a large amount of training data for calibration. In addition, the reduced complexity facilitates error detection and fine-tuning. For our approach, the estimation error was low at low hair densities and negligible at hair densities >5%, guaranteeing high reproducibility. There was an almost perfect match of real hair density values and calculated hair density values, indicating the high reliability of the calculated percent values of hair densities.

The validation of the effectiveness of the monitoring methods in the field trial showed a robust correlation between the results of the hair tubes and the results of the wildlife cameras across the three habitats considered. This is in line with findings for larger vertebrate species, such as brown bears (*Ursus arctos*) [41], European minks (*Mustela lutreola*) [42], and grey squirrels (*Callosciurus erythraeus*) [43]. In this study, hair from hair tubes was utilized as a reflection of the small mammal community, which may be too broad if particular target species or even individuals are in focus. There may be some bias from counting the same individual repeatedly but if and to what extent this was present is unclear and could be assessed in future studies that combine hair tubes and/or cameras with PIT-tag readers.

The combination of wildlife cameras as another indirect method and hair tubes seems appropriate for identifying species, if frequent checks of hair tubes can be conducted. Genetic information can be extracted from hair via molecular analyses [14], and the morphological assessment of species is possible but may be too costly or for other reasons impossible. If the genetic or morphological identification of species or the genetic identification of individuals from hair is an option, hair tubes can yield meaningful data at a much finer resolution, such as that in the community approach we applied.

The advantages of hair tubes are their low costs, simple construction, ease of operation, moderate field sampling effort, and potential to be used at any spatial scale [5,6]. The use of hair tubes is particularly suitable for rare, elusive, and cryptic small mammals as well, as endangered species must not be disturbed, let alone harmed [13,44]. Hair tubes may be particularly useful in settings where mostly one species occurs such as common voles (*Microtus arvalis*) in European grasslands [45] and house mice (*Mus musculus*) in Australian wheat fields [46] because in the absence of other small mammals, hair tubes work on the species level. 

With varying hair tube designs and baits as well as placements, different species of small mammals can be monitored with hair tubes. For example, hair tubes were used to target the wood mouse (*Apodemus sylvaticus*) at different shrub layer heights and confirmed that this species not only moves at the ground level, but also a few meters above the ground [47]. Hair tubes may also support the monitoring of buildings to detect commensal rodents such as Norway rats (*Rattus norvegicus*) and house mice (*Mus musculus*) early and to initiate timely action. This would be highly beneficial for rodent pest control. In this context, monitoring with hair tubes may also offer the opportunity to identify whether target species (Norway rats; house rats (*Rattus rattus*), house mice] or non-target species are present, by analyzing hair samples.

Non-invasive techniques as indirect methods to study the activity of small mammals are a valuable tool, as they reduce the disturbance and potential harm caused to animals when invasive methods are used [13]. Hair tubes are such a non-invasive method, suitable for settings where direct contact is not required to study the ecology of small mammals.

## 5. Conclusions

The program developed in this study enables the semi-automated quantitative analysis of hairs on adhesive tape to more precisely determine the activity of small mammals based on hair tubes. Hair tubes reliably detect small mammal activity in various habitats—more correctly in hedges (correlation coefficient = 0.79) than in grasslands (correlation coefficient = 0.43). The technique represents an easy-to-use, non-invasive method for the determination of small mammal activity in suitable settings. The estimation error is small when hair density is low and negligible at hair densities >5%, guaranteeing high reproducibility. The method should be validated in further field trials for other habitats and species compositions, optimally for a wide range of hair densities. The rapid development of genetic methods may allow easier and cheaper species-based or even individual-based analyses of hair from hair tubes to be conducted in the years to come. 

## Data Availability

Original data are available in Appendix A.

## Appendix A

**Table A1:** Data used for Figure 3 showing the correlation of real and calculated hair density values. Percent values are real and calculated hair density values per replicate (xxxxxx real (codes for real values) = real value; the following 9 replicates (codes for calculated values) = corresponding replicates of calculated values).

ID	Replicate	Percent									
1	B5R8Ha real	0.07	36	B5R9Ha 6	4.44	71	A2I7Ha real	12.77	106	D2I2Ga 6	17.23
2	B5R8Ha 2	0.15	37	B5R9Ha 7	4.58	72	A2I7Ha 2	12.93	107	D2I2Ga 7	17.22
3	B5R8Ha 3	0.16	38	B5R9Ha 8	3.96	73	A2I7Ha 3	12.85	108	D2I2Ga 8	17.24
4	B5R8Ha 4	0.15	39	B5R9Ha 9	4.55	74	A2I7Ha 4	13.01	109	D2I2Ga 9	17.25
5	B5R8Ha 5	0.16	40	B5R9Ha 10	4.18	75	A2I7Ha 5	12.8	110	D2I2Ga 10	17.23
6	B5R8Ha 6	0.25	41	A2I3Hb real	3.19	76	A2I7Ha 6	13.15	111	B5I9Ha real	18.21
7	B5R8Ha 7	0.25	42	A2I3Hb 2	3.32	77	A2I7Ha 7	13.07	112	B5I9Ha 2	18.32
8	B5R8Ha 8	0.28	43	A2I3Hb 3	3.28	78	A2I7Ha 8	13.08	113	B5I9Ha 3	18.38
9	B5R8Ha 9	0.25	44	A2I3Hb 4	3.29	79	A2I7Ha 9	13.11	114	B5I9Ha 4	18.31
10	B5R8Ha 10	0.26	45	A2I3Hb 5	3.3	80	A2I7Ha 10	13.09	115	B5I9Ha 5	18.37
11	D3I2Gb real	0.25	46	A2I3Hb 6	4.61	81	A2R1Ha real	14.26	116	B5I9Ha 6	18.49
12	D3I2Gb 2	1.67	47	A2I3Hb 7	4.58	82	A2R1Ha 2	14.07	117	B5I9Ha 7	18.42
13	D3I2Gb 3	1.71	48	A2I3Hb 8	4.65	83	A2R1Ha 3	14.4	118	B5I9Ha 8	18.59
14	D3I2Gb 4	1.74	49	A2I3Hb 9	4.62	84	A2R1Ha 4	14.5	119	B5I9Ha 9	18.84
15	D3I2Gb 5	1.73	50	A2I3Hb 10	4.62	85	A2R1Ha 5	14.27	120	B5I9Ha 10	18.59
16	D3I2Gb 6	1.74	51	D1R3Ga real	4.36	86	A2R1Ha 6	14.05			
17	D3I2Gb 6	1.73	52	D1R3Ga 2	4.52	87	A2R1Ha 7	13.97			
18	D3I2Gb 7	1.74	53	D1R3Ga 3	4.39	88	A2R1Ha 8	14.47			
19	D3I2Gb 8	1.74	54	D1R3Ga 4	4.54	89	A2R1Ha 9	14.17			
20	D3I2Gb 9	1.74	55	D1R3Ga 5	4.42	90	A2R1Ha 10	14.34			
21	B2R8Ha real	1.35	56	D1R3Ga 6	4.77	91	D1I1Gb real	14.47			
22	B2R8Ha 2	1.53	57	D1R3Ga 7	4.64	92	D1I1Gb 2	14.56			
23	B2R8Ha 3	1.57	58	D1R3Ga 8	4.76	93	D1I1Gb 3	14.6			
24	B2R8Ha 4	1.58	59	D1R3Ga 9	4.8	94	D1I1Gb 4	14.76			
25	B2R8Ha 5	1.59	60	D1R3Ga 10	4.88	95	D1I1Gb 5	14.66			
26	B2R8Ha 6	1.7	61	A2R6Hb real	8	96	D1I1Gb 6	14.35			
27	B2R8Ha 7	1.7	62	A2R6Hb 2	7.92	97	D1I1Gb 7	14.56			
28	B2R8Ha 8	1.67	63	A2R6Hb 3	7.5	98	D1I1Gb 8	14.53			
29	B2R8Ha 9	1.71	64	A2R6Hb 4	7.93	99	D1I1Gb 9	14.27			
30	B2R8Ha 10	1.7	65	A2R6Hb 5	8.11	100	D1I1Gb 10	14.47			
31	B5R9Ha real	3.15	66	A2R6Hb 6	7.95	101	D2I2Ga real	17.41			
32	B5R9Ha 2	3.26	67	A2R6Hb 7	7.59	102	D2I2Ga 2	17.64			
33	B5R9Ha 3	3.31	68	A2R6Hb 8	7.74	103	D2I2Ga 3	17.49			
34	B5R9Ha 4	3.26	69	A2R6Hb 9	7.93	104	D2I2Ga 4	17.53			
35	B5R9Ha 5	3.28	70	A2R6Hb 10	8.08	105	D2I2Ga 5	17.55			

**Table A2:** Number of sections of adhesive tape with and without hair per habitat.

Habitat	With Hair	Without Hair	% With Hair
Wheat	349	449	43.7
Hedge	379	421	47.4
Grassland	358	444	44.6

**Table A3:** Sightings from wildlife camera pictures of small mammals per taxon and habitat.

Habitat	Species	
	*Apodemus*	Voles
Wheat	389	20
Hedge	431	95
Grassland	400	78

**Table A4:** Data used for Figure 4: correlation between hair tube results and wildlife camera results.

ID	Camera	Tube	Habitat												
1	4	0.00	Grassland	37	4	0.14	Hedge	73	52	2.30	Hedge	109	55	1.42	Wheat
2	11	0.00	Grassland	38	0	0.00	Hedge	74	0	0.02	Hedge	110	6	1.39	Wheat
3	5	0.00	Grassland	39	0	0.00	Hedge	75	6	1.16	Hedge	111	50	1.59	Wheat
4	0	0.00	Grassland	40	0	0.03	Hedge	76	3	0.22	Hedge	112	0	0.58	Wheat
5	0	0.00	Grassland	41	68	3.36	Hedge	77	0	0.00	Hedge	113	42	0.88	Wheat
6	0	0.00	Grassland	42	16	2.70	Hedge	78	11	1.82	Hedge	114	2	0.01	Wheat
7	0	0.02	Grassland	43	29	0.88	Hedge	79	18	2.05	Hedge	115	15	0.71	Wheat
8	0	0.00	Grassland	44	33	1.86	Hedge	80	4	0.56	Hedge	116	1	0.19	Wheat
9	26	1.40	Grassland	45	14	0.94	Hedge	81	10	0.23	Hedge	117	28	1.00	Wheat
10	2	1.29	Grassland	46	16	3.10	Hedge	82	7	0.00	Wheat	118	0	1.16	Wheat
11	8	1.07	Grassland	47	10	0.47	Hedge	83	0	0.00	Wheat	119	0	2.58	Wheat
12	8	1.71	Grassland	48	8	0.30	Hedge	84	1	0.00	Wheat	120	0	0.51	Wheat
13	40	2.18	Grassland	49	28	2.33	Hedge	85	7	0.07	Wheat				
14	0	2.11	Grassland	50	44	1.60	Hedge	86	9	0.00	Wheat				
15	5	0.26	Grassland	51	20	2.13	Hedge	87	2	0.00	Wheat				
16	10	0.44	Grassland	52	4	1.59	Hedge	88	1	0.00	Wheat				
17	29	0.48	Grassland	53	13	1.90	Hedge	89	50	3.99	Wheat				
18	41	0.64	Grassland	54	22	0.73	Hedge	90	15	0.65	Wheat				
19	0	2.37	Grassland	55	8	1.90	Hedge	91	0	0.00	Wheat				
20	0	0.48	Grassland	56	8	1.64	Hedge	92	0	0.00	Wheat				
21	0	0.03	Grassland	57	15	2.00	Hedge	93	6	2.67	Wheat				
22	18	1.23	Grassland	58	3	0.07	Hedge	94	16	2.30	Wheat				
23	7	0.97	Grassland	59	7	1.17	Hedge	95	0	0.72	Wheat				
24	2	0.30	Grassland	60	2	0.00	Hedge	96	7	0.10	Wheat				
25	19	0.15	Grassland	61	9	0.41	Hedge	97	2	0.24	Wheat				
26	1	0.00	Grassland	62	0	0.00	Hedge	98	27	1.99	Wheat				
27	0	0.00	Grassland	63	5	1.56	Hedge	99	0	0.40	Wheat				
28	5	0.02	Grassland	64	11	0.00	Hedge	100	0	1.18	Wheat				
29	0	5.13	Grassland	65	29	3.81	Hedge	101	27	1.70	Wheat				
30	25	2.68	Grassland	66	0	0.00	Hedge	102	4	0.13	Wheat				
31	26	2.03	Grassland	67	28	1.33	Hedge	103	5	0.10	Wheat				
32	19	0.46	Grassland	68	0	0.00	Hedge	104	0	0.00	Wheat				
33	56	1.45	Grassland	69	3	0.73	Hedge	105	13	0.60	Wheat				
34	15	0.91	Grassland	70	0	0.00	Hedge	106	1	0.63	Wheat				
35	33	2.98	Grassland	71	17	0.11	Hedge	107	11	0.07	Wheat				
36	12	0.99	Grassland	72	0	0.00	Hedge	108	0	0.00	Wheat				
